# Improving Spiritual Well-Being of Polish Pain Outpatients: A Feasibility Mixed Methods Study

**DOI:** 10.3390/jcm13123615

**Published:** 2024-06-20

**Authors:** Maciej Wiktor Klimasiński, Ewa Baum, Katarzyna Wieczorowska-Tobis, Bogusław Stelcer

**Affiliations:** 1 Katedra Medycyny Paliatywnej, Uniwersytet Medyczny w Poznaniu, 61-701 Poznań, Poland; tobis@ump.edu.pl; 2 Katedra Nauk Społecznychi Humanistycznych, Uniwersytet Medyczny w Poznaniu, 61-701 Poznań, Poland; ebaum@ump.edu.pl; 3 Katedra Psychologii Klinicznej, Uniwersytet Medyczny w Poznaniu, 61-701 Poznań, Poland; stelcer@ump.edu.pl

**Keywords:** interpersonal relations, spirituality, chronic pain, religion and medicine

## Abstract

**Introduction**: A physician in a chronic pain treatment clinic must recognize that the relationship between pain and spirituality is bidirectional. Chronic pain can decrease the level of spiritual well-being, and low spiritual well-being can also significantly intensify the perception of pain and worsen coping with it. Currently, for many scientific and medical communities, it is evident that spiritual care is an indispensable element of holistic medicine. **Objective**: The authors developed a non-religious spiritual care model provided by a physician at a chronic pain treatment clinic from May 2022 to February 2024. **Method**: The study utilized a mixed-method approach to conduct the research. The analysis consisted of twelve patients. A FACIT-Sp-12 questionnaire evaluated the individual’s spiritual well-being before the intervention. The intervention involved asking patients open-ended questions about their life history, experiences, and spiritual beliefs and the physician’s use of active listening and empathetic responses to what patients shared (relationship-building activities). The intervention aimed to assist patients in accepting the limitations of an incurable chronic disease, affirming the value of their lives, enhancing inner harmony, and increasing their sense of belonging to something greater. After the intervention, a re-assessment of the patient’s spiritual well-being was conducted using the FACIT-Sp-12 questionnaire. Researchers collected qualitative data through a confidential survey that included the following instructions: “Please express an anonymous opinion on how you perceive the spiritual care provided by the physician”. **Results**: There was an increase in spiritual well-being, assessed using the FACIT-Sp-12 scale, in 9 out of 12 patients. The median, as well as the average, level of spiritual well-being increased in a statistically significant way after the intervention (*p* < 0.05). This was primarily due to the higher value of the peace subscale of the questionnaire. Qualitative analysis revealed benefits reported by patients (personal development, gratitude, satisfaction, support, hope) resulting from physician’s actions. **Conclusions**: Both qualitative and quantitative data showed that establishing a relationship with the doctor improves the spiritual well-being of patients. Therefore, this model can be recommended for physicians in chronic pain treatment clinics.

## 1. Introduction

Progressive chronic illness can significantly impact patients’ spirituality. The authors discussed the definition of spirituality developed by the Polish Association for Spiritual Care in Medicine in a previous publication [1]. Chronic pain often negatively affects broadly understood spirituality—the interpretation of the meaning and purpose of patients’ life, their internal harmony and moral stance, and how they experience relationships with other people [1,2]. Meanwhile, referring to transcendence (the human relation to what surpasses them) and the ability to go beyond one’s limitations may prove crucial in adapting to living with chronic pain [1,3]. The relationship between pain and spirituality is, therefore, bidirectional, as chronic pain can decrease the level of spiritual well-being and the lack of spiritual well-being can worsen the symptoms of chronic diseases, particularly the level of pain experienced [4]. One study conducted in Poland assessed the self-reported levels of religiosity and spirituality among chronically ill patients. Almost all patients identified as Catholics; 54.7% reported that faith was a resource for coping with suffering, and 38% reported participating in religious meetings. The study showed that spiritual distress correlates not with the level of religiosity but with the intensity and duration of the illness [5]. Therefore, this concern applies to everyone who suffers from a severe and long-term illness, regardless of whether they are religious or not [6,7]. 

According to literature reviews, patients want to discuss spirituality during medical consultations [8,9,10,11]. Moreover, patients whose spiritual needs are unmet report lower quality of life and satisfaction with medical care [12,13,14,15]. Currently, for many scientific and medical communities, it is evident that spiritual care is an essential element of holistic medicine. According to various expert panels and organizations issuing guidelines for medical professionals, physicians should have basic competencies in spiritual care and routinely incorporate spiritual support into the care of patients with serious illnesses [6,11,14,16,17,18,19,20,21,22]. Even though, recently there was an explosion of research in this field, professionals still need help understanding how to move beyond the initial questions of whether spirituality and/or religion are important to the patient and/or their religious preferences [23]. Meanwhile, in many countries, there are already courses on spirituality for medical students and models of spiritual support to complement standard treatment protocols in medical facilities [24,25,26,27].

Both patients and physicians agree that chronic pain, as a multidimensional phenomenon, requires consideration of the spiritual dimension in its treatment. Still, medical professionals highlight difficulties addressing these issues [28,29]. Numerous reviews of spiritual interventions in the treatment of both cancer pain [30,31] and non-cancer pain (headaches, musculoskeletal pain) [32,33,34,35] are available; however, to the best of the authors’ knowledge, a model of spiritual care in the chronic pain treatment clinic has not been published so far. According to Zieniuk, a human connection with a doctor can motivate and strengthen chronic pain patients who perceive the course of events more as harm, misfortune, failure, or threat than a challenge [36]. This study aims to introduce a newly developed model of non-religious spiritual care based on a positive relationship with a physician.

## 2. Materials and Method

### 2.1. Organization of the Study 

This study, lasting from May 2022 to February 2024, evaluated a spiritual care model at a chronic pain clinic using a mixed-method approach. As a feasibility study without a control group, the research focused on a small patient cohort at a pain treatment clinic in Poznań, Poland. Inclusion criteria were age over 18 and moderate to severe chronic pain lasting more than six months. Patients with active cancer were excluded. Participants received no compensation for their involvement.

Basic demographic data were collected. Initially, patients provided written consent and underwent an assessment of their spiritual well-being using the FACIT-Sp-12 questionnaire. A month later, the intervention was applied, followed by another evaluation after another 30–60 days to measure spiritual well-being changes using the FACIT-Sp-12 questionnaire. Later on, participants provided feedback on the model through anonymous surveys. The Bioethics Committee at K. Marcinkowski Medical University approved the study on 17 February 2022 (Resolution No. 112/22).

### 2.2. Intervention

The goal of spiritual care is to alleviate spiritual distress and enhance spiritual well-being. This involves helping patients accept the limitations imposed by incurable chronic diseases, affirming the meaning of life, and fostering internal harmony and a sense of connectedness to a greater whole. It is suggested that all chronic pain patients, particularly those with low scores on the FACIT-Sp-12 scale, should undergo interventions aimed at improving their spiritual well-being.

Therefore, a non-religious spiritual intervention was developed in three phases: analyzing the non-religious aspects of spirituality, assessing the spiritual needs of Polish non-religious individuals, and conducting a literature review of existing spiritual care models. Themes of empathy, relationship building, and showing interest in patients’ stories and spiritual experiences emerged. Based on this, an original Spiritual Intervention Guide was created, which involves a conversation with the patient about their life history, spiritual experiences, and beliefs (Table 1). It combines the advantages of both a structured and open approach. It facilitates flexible discussion by using essential questions that can be replaced with auxiliary questions. Crucially, the intervention incorporates relationship-building activities performed by the doctor, such as active listening (paying attention to the patient’s non-verbal communication and paraphrasing) and responding empathetically to patients’ difficult confessions. The honest answers to these intimate questions were validated and appreciated but not recorded. The entire intervention took around 20 min to conduct. 

### 2.3. Tool

The original developers have created the FACIT-Sp-12 questionnaire to assess an individual’s spirituality briefly and concisely. This tool has been used in numerous studies, translated and validated in Polish [37]. It is a self-report tool comprising 12 items rated on a 5-point Likert scale. The questionnaire has three subscales measuring individual aspects of spiritual well-being: peace, meaning, and faith. The total score for spiritual well-being was calculated by summing the scores of each of the three subscales. In cases where individual items are skipped, subscale scores can be prorated using the average of the other answers in the scale. This is acceptable as long as more than 50% of the items were answered in the subscale [38].

The second research method involved giving each patient a confidential survey with the following instruction: “Please express your anonymous opinion on how you perceive the spiritual care provided by the doctor”.

### 2.4. Data Analysis

Statistical analyses of the quantitative part of the study were conducted using STATISTICA 13.0 software (StatSoft, Kraków, Poland). The data from the FACIT-Sp-12 questionnaire were presented as medians and ranges, reflecting non-normal distribution. The Shapiro–Wilk test was utilized to assess the normality of the data distribution. The Wilcoxon test was employed to compare two paired data sets. Also the effect size was calculated. A *p*-value of less than 0.05 was deemed statistically significant.

Regarding the qualitative part of the study, two researchers (BS and KWT) independently analyzed participants’ responses to confidential surveys. They examined the data to identify recurring themes. This process led to identifying quotes, which each researcher associated with themes they identified earlier. Subsequently, these themes were compared during a meeting, and a discussion ensued, resulting in a consensus on four key themes.

## 3. Results

Between May 2022 and February 2024, 13 patients completed the study, including ten women and three men. One participant had to be excluded from the study because they failed to answer 50% of the questions in one of the subscales. The average age was 61.2 years (min: 30 years, max: 78 years). Diagnoses made by the referring physicians to the clinic were M79.7 (fibromyalgia); M15 (polyosteoarthritis); M51.1 (lumbosacral intervertebral disc disorders with radiculopathy); G58 (other mononeuropathies); M54 (chronic back pain); M32 (systemic lupus erythematosus); M47 (spondylosis); G54 (lumbosacral root disorders); M45 (ankylosing spondylitis); G62 (other polyneuropathies). The median level of the spiritual well-being of patients, assessed by the FACIT-Sp-12 questionnaire before the intervention, was 31.8 (range: 10–40), and after the intervention, it was 32.3 (range: 14–43; *p* < 0.05). This was mainly due to the higher value of the peace subscale: before the intervention, it was 8.5 (range: 2–9), and after the intervention, it was 10.8 (range: 2–13, *p* < 0.05).

**Figure 1:**
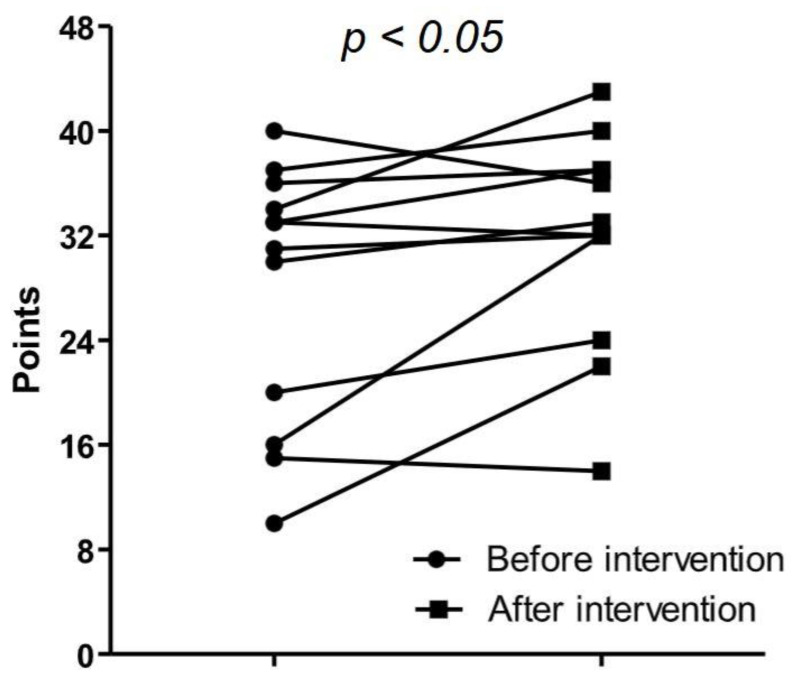
The individual data for the total FACIT-Sp-12 questionnaire score performed twice for all participants.

The remaining subscales’ results were comparable (meaning: 12.5; range: 4–14 vs. 12.5; range 6–16; and faith: 9.0; range: 2–16 vs. 9.5; range: 5–16). The individual data for the total FACIT-Sp-12 questionnaire, performed twice for all participants, are shown in Table 2 and Table 3 and Figure 1. After the intervention, the results were better in 9 individuals. There were two spectacular improvements (in one subject of 12 points and another even of 16). The median improvement was 4.0 (range: 1–16). Three patients’ scores on the FACIT-Sp-12 questionnaire were lower after the intervention. Among them, the results of the second assessment of two patients differed by only 1 point. In the third patient, the worsening was more severe (4 points).

Qualitative results, translated into English, are presented in Table 4. Ten patients returned the confidential surveys. Their opinions detailed their interactions with the healthcare provider and the impact of those interactions on their well-being. The responses made it possible to identify four recurring themes, which are illustrated by selected quotes in Table 4. Those themes were personal development; sense of gratitude; satisfaction with relationship quality; feeling of support; sense of hope. All opinions emphasized the significance of the doctor’s approachability, empathy, and attentiveness. Patients expressed satisfaction and gratitude with the way the doctor conducted the conversations. They appreciated the non-restrictive atmosphere of emotional safety and non-judgmental understanding. They felt valued and treated as individuals, not just cases. Accompaniment by the doctor in coping with the hardships of the disease was found to strengthen psychological resources as well as bring hope for managing symptoms and having an acceptable level of quality of life. The magnitude of the impact varied. Some patients expressed significant pain relief and functional improvement as well as reduced anxiety and depression, while others less so. 

## 4. Discussion

In this pilot study, we applied our newly developed model of spiritual care to enhance the spiritual well-being of patients with chronic pain via a non-religious spiritual intervention conducted by a pain clinic physician. We considered this a physician’s task because low spiritual well-being tends to exacerbate physical pain [39,40]. The intervention consisted of a semi-structured, in-depth conversation exploring the patient’s life history, unique spiritual experiences, alongside the physician’s use of active listening and empathetic responses (relationship-building activities). We found that posing a question encouraging patients to discern a deeper meaning in life amidst illness could trigger feelings of guilt in those unprepared, especially when asked by an authoritative figure. Consequently, this auxiliary question was removed from our Spiritual Intervention Guide. The level of spiritual well-being, as assessed by a validated tool, increased significantly, and qualitative data demonstrated the “breakthrough” value of spiritual care.

It was not solely the single 20-min Spiritual Intervention that was influential. These patients met a physician who recommended adjustments to the pain management regimen and also demonstrated empathy and built a rapport by expressing genuine concern for their experience of chronic pain. In subsequent visits, the physician engaged more deeply by exploring how the illness impacted the patient’s life and discussing their spiritual experiences. Some patients were also qualified for pain-blocking interventions, which involved additional 30-min sessions with the doctor. Further assessments of spiritual well-being post-intervention were conducted in the following visits, and subsequent appointments gathered anonymous feedback on the spiritual care provided. The cumulative duration of the therapeutic relationship with the physician could extend up to six months. Throughout this period, patients could discover their own resilience in combating the illness, facilitated by open conversations with a supportive figure and joint exploration of their personal resources. The clinic’s patients expressed high satisfaction with such a formula of spiritual care. However, this study does not establish the clinical efficacy of the intervention due to the absence of a control group. The next phase after this exploratory study will be a randomized trial with a bigger cohort. It should take more variables into account, like the exact amount of time since the diagnosis to exclude changes in spiritual wellbeing due to ongoing patient adaptation to their conditions.

The issue under discussion is not a leading topic of research in medicine; there are individual studies on the relationships between pain, various aspects of spirituality, and the doctor–patient relationship. Huperz et al. conducted a study that examined the acceptability, feasibility, and potential benefits of doctors conducting interviews about patients’ spiritual histories. Similarly, they found that these conversations significantly improve patient and family doctors’ relationships. The authors concluded that this relationship positively impacts health (including less pain severity) [41]. Assing Hvidt et al. developed a tool named EMAP, which also aimed to enhance the existential well-being of the patient and help deepen the relationship between the family doctor and the patient [42]. Ellingsen et al. simultaneously assessed brain activity (fMRI hyperscanning) in patients with chronic pain and clinicians during live interactions and found that empathy and support can reduce pain intensity [43]. All of this confirms the findings of the study conducted by Selman and colleagues that the quality of spiritual care depends on the quality of the relationships established, not on the nature of the intervention itself [11]. Our study did not aim to demonstrate the extent to which spiritual intervention reduced the level of pain experienced. We were unable to assess this because, at the same time, all participants also had their pharmacological pain management modified and/or they underwent pain interventions performed by an anesthesiologist. This is an area that needs improvement in the planning of future studies. 

The impact of other types of non-religious spiritual interventions on levels of spiritual well-being, spiritual distress, and perceived pain has been studied and described. Muehlhausen et al. evaluated the effect of 4 sessions with a chaplain who did not represent any specific religious denomination. Similarly to our intervention, the chaplain’s most common actions were active listening and showing care and interest. A study on twenty patients revealed decreased religious or spiritual distress and increased overall well-being [44]. Afrasiabifar et al. developed their intervention and conducted a controlled randomized study, which showed a significant improvement in the spiritual well-being of 37 cancer patients. The sessions conducted by a nurse included expressing personal experiences, interactive negotiation, mutual questions and answers, presentation of short audio and video clips, discussion of a book, and handing out a brochure [45].

The relational aspect of spirituality (connectedness) is generally recognized as inseparable and has been described by the authors in another publication [1]. Healthcare providers should build their relationship with patients because relationships have the potential to foster hope, especially the kind hope that goes beyond goal attainment [46]. Another tool from the FACIT group, namely FACIT-Sp-Ex, contains the same 12 questions as FACIT-Sp-12, plus a few additional questions about relationships and connectedness, e.g., “I feel loved” [37]. If chronic pain causes spiritual distress in a patient that is mainly related to feeling misunderstood or “unloved”, then it seems that establishing a therapeutic relationship with the doctor can effectively reduce these symptoms. By respecting the patient and getting to know them better, the doctor can strengthen their ability to cope with pain. Another suggestion for future studies is to consider using the FACIT-Sp-Ex instead of the FACIT-Sp-12 tool.

## 5. Conclusions

The introduction of the “Non-Religious Spiritual Care Model in the Pain Clinic” discussed in this study may require some physicians to change their perspective from the traditional “treatment of diseases” to “caring for the suffering individual”. Patients have unique life stories, emotional responses, and social relationships. They are also individuals grappling with questions about the meaning and purpose of their lives in the face of gradually losing their physical ability. Patient care involves being interested in patients’ experiences, which include understanding, empathy, kindness, and respect for their values. These experiences can be equally termed “holistic care”, “patient-centered care”, or “compassionate care”. Our study does highlight a perspective on spiritual care that emphasizes the quality of interpersonal interactions, a genuine human-to-human connection. This can lead patients to see their situation from a new perspective, enabling them to accept their limitations, move beyond their usual coping methods, and find a new meaning in life. Such relationships, which may develop, benefit both parties, as love expressed through care, support, empathy, and openness is needed in everyone’s life. This is why the role and significance of spirituality are invaluable in medicine.

## Figures and Tables

**Table 1:** Authors’ Original Non-Religious Spiritual Intervention Guide.

**Introduction to Part 1**
From the interview I conducted with you, it is evident that chronic pain significantly affects your: functionality/occupational performance/leisure activities/mood/social life (please cross out the irrelevant). I would like to understand better how pain influences your life, so I will ask you about your life history. I will not judge your answers in any way.
**Main Question of Part 1**
Please talk about your past and how your life has changed due to your illness.
**Auxiliary Questions for Part 1**
What was the most important event in your life?
How would you describe the meaning of your life?
How do you interpret your illness/what deeper meaning might it have?
How has the illness affected your attitude towards life?
**Introduction to Part 2**
For some people, spiritual experiences and beliefs significantly impact how they experience and cope with chronic illness. I would like to know how they affect you. I fully respect that each person experiences their spirituality in their own unique way (diversity or lack of religious affiliation).
**Main Questions of Part 2**
How would you describe your spiritual life/spiritual world? Has anything changed in this area because of your illness?
**Auxiliary Questions for Part 2**
What, in your opinion, is spirituality?
How does spirituality affect your experience of illness?
How do you express your spirituality?
Do you participate in any spirituality-related meetings?
Do any specific beliefs or spiritual activities help you cope with pain?
How could you deepen your spirituality?

**Table 2:** The individual data for the total FACIT-Sp-12.

FACIT-Sp-12 before Intervention	Meaning Subscale	Peace Subscale	Faith Subscale	Total Score	FACIT-Sp-12 after Intervention	Meaning Subscale	Peace Subscale	Faith Subscale	Total Score
Patient 1	13	9	11	33	Patient 1	15	8	9	32
Patient 3	12	12	16	40	Patient 3	12	10	14	36
Patient 4	12	9	16	37	Patient 4	13	11	16	40
Patient 5	14	8	9	31	Patient 5	13	12	7	32
Patient 6	10	2	3	15	Patient 6	7	2	5	14
Patient 7	8	7	5	20	Patient 7	10	8	6	24
Patient 8	13	10	13	36	Patient 8	14	11	12	37
Patient 9	14	8	8	30	Patient 9	12	11	10	33
Patient 10	4	4	8	16	Patient 10	11	11	10	32
Patient 11	4	4	2	10	Patient 11	6	8	8	22

**Table 3:** Statistical analyses.

	Before Intervention		After Intervention				
	Mdn	IQR	Mdn	IQR	Z	*p*	η^2^
Meaning 2—Meaning 1	12.50	5.25	12.50	4.5	−1.30	0.193	0.07
Peace 2—Peace 1	8.500	5	10.83	3.75	−2.36	**0.018**	0.23
Faith 2—Faith 1	9.00	7	9.50	6.25	−0.99	0.321	0.04
Total 2—Total 1	31.750	18.5	32.33	11	−2.13	**0.034**	0.19

A *p*-value of less than 0.05 has been put in bold to show statistical significance.

**Table 4:** Analysis of patients’ opinions regarding patients’ experiences.

**Theme 1: Personal development**
Patient No. 1: The doctor has opened many doors for me that I had never looked into before. He showed me how I can be strong despite the great pain in my body and mind.
Patient No. 3: I was very satisfied with the spiritual care provided by the doctor. I want to continue the meetings (…) to strengthen my peace of mind and control over pain.
Patient No. 8: The conversations with my attending doctor are very beneficial to me.
Talking with the doctor has a good impact on my mental and spiritual health.
**Theme 2: Satisfaction with the quality of relationship**
Patient No. 4: He always listened to what hurts and how. He was genuinely interested in how to help.
Patient No. 4: Even talking to the doctor brings me relief; I do not think about the pain.
Patient No. 7: …he treated me like a person, not an old creaky woman.
**Theme 3: Sense of support**
Patient No. 4: The doctor understands me very well and does everything possible to help me.
The doctor will help me, and together we will fight as long as I have the strength so that my spiritual well-being is as good as possible.
Patient No. 5: To say that God sent me the doctor I now visit, who supports me greatly! Showing interest and understanding means so much and provides immense support.
His approach to my health problem is like a balm to the heart, a great support.
**Theme 4: Sense of hope**
Patient No. 4: The first meeting already gave me hope (…) each meeting gives me hope. (…) I always come with hope for the next meetings and conversation.
Patient No. 5: My life, my journey with illnesses, has a different dimension now. I look forward to the visits…

## Data Availability

All the raw data is included in the article.
